# Nano-enabled enhancement of salt stress tolerance in barley using chitosan-selenium nanoparticles: physiological and molecular insights

**DOI:** 10.1038/s41598-026-41850-3

**Published:** 2026-03-03

**Authors:** Fatemeh Gholizadeh, Zahra Tahmasebi, Tibor Janda

**Affiliations:** 1 Department of Plant Physiology and Metabolomics, Agricultural Institute, HUN-REN Centre for Agricultural Research, Martonvásár, 2462 Hungary; 2 Festetics Doctoral School, MATE Hungarian University of Agricultural and Life Sciences, Keszthely, Hungary

**Keywords:** Barley, Chitosan-selenium nanoparticles, Gene expression, Salt stress, Agricultural genetics, Plant physiology

## Abstract

Salinity stress severely limits barley (*Hordeum vulgare* L.) growth and productivity. This study examined the effects of chitosan (Cs), selenium (Se), and chitosan-selenium nanoparticles (Cs-Se NPs) on salt tolerance of two barley cultivars, Mv Initium and Tectus, exposed to 0, 100, and 200 mM NaCl. Salinity reduced plant height, biomass, and chlorophyll content. Foliar application of Cs and especially Cs-Se NPs significantly improved these traits. Cs-Se NPs enhanced proline (PRO) accumulation and activities of ascorbate peroxidase (APX) and catalase (CAT) under salt stress in both cultivars, which supports improved ROS scavenging capacity. The significant upregulation of antioxidant enzyme genes (*HvAPX*, *HvSOD*, *HvCAT*) following Cs-Se NPs treatment under salinity strongly indicates enhanced reactive oxygen species (ROS) detoxification. Key ion homeostasis genes (*HvSOS1*, *HvSOS3*, *HvNHX1* and *HvHKT2*) were also upregulated, supporting improved salt stress tolerance. Strong correlations were found between antioxidant activity, chlorophyll content, and growth. These findings suggest that Cs-Se NPs effectively boost barley’s physiological and molecular defenses against salinity.

## Introduction

Barley (*Hordeum vulgare L.*) is recognized as the fourth most economically significant cereal crop in the world in terms of production volume and cultivated area^1^. This crop serves as a healthy dietary source, rich in fiber and bioactive compounds, and is primarily used for animal feed and industrial purposes^2^. Barley grows across a wide range of geographical and climatic conditions^3^, highlighting its high genetic diversity^4^. Similar to many other agricultural crops, environmental stresses cause substantial crop loss worldwide, therefore protection plants against them, and better understanding the defense mechanisms are of great importance. Barley’s growth, production, and yield are especially vulnerable to environmental factors such as soil salinity, which can severely impact its overall performance^5–7^. Salinity is one of the most significant and prevalent abiotic stresses, rendering approximately one-third of the worlds arable lands unsuitable for agriculture. Consequently, about 20% of agricultural lands are currently affected by salinity stress^8–10^. Salinity causes both ionic and osmotic stresses, leading to nutrient imbalance, disrupted cellular ion homeostasis, and oxidative stress^11–13^. All growth processes in plants, from seed germination to yield production, are negatively impacted by high soil salt content^14^. Despite these challenges, barley is known as a crop with notable tolerance to saline conditions^15,16^. Barley employs diverse and complex regulatory pathways to tolerate salinity stress. These mechanisms include water uptake regulation, photosynthesis modulation, hormonal signaling, antioxidant defense systems, osmotic balance maintenance through the synthesis of osmoprotectants, and ionic homeostasis. These pathways involve the detection and signaling of salinity, as well as the identification of genes responsive to salt stress, which serve as valuable sources of salt-tolerance alleles^5,17^.

According to the findings of Schwarz and Foltz, selenium (Se) is an essential element for the growth of living organisms. Se is also a beneficial nutrient for plants, enhancing their growth and yield, while exhibiting antioxidant activities^18–20^. At low concentrations, Se promotes plant growth, maintains mitochondrial integrity; however, at high concentrations, it may lose its beneficial effects^21,22^.

A study by Diao et al. revealed that Se reduces damage to photosynthesis in tomato plants under salt stress. Additionally, Se has a positive effect on photosynthesis and antioxidant defense systems^23,24^. When applied as a spray, Se enhances growth, increases proline content, and boosts photosynthetic pigments in cucumbers under salt stress, thereby improving salinity tolerance. Se also increases the concentration of secondary metabolites in plants. For instance, in (*Brassica juncea L.*) it induces the expression of the phenylalanine ammonia lyase (*PAL*) gene and promotes the production of secondary metabolites^25^.

Nanoparticles (NPs) are excellent carriers for the delivery of drugs and various compounds to organisms, including plants, and have emerging applications in enhancing salinity tolerance in plants^26^. It has been observed that nanoparticles increase growth and antioxidant content in medicinal plants under salt stress^27^. Another study demonstrated that Se nanoparticles improved antioxidant activity in groundnut plants under stress conditions^28^. Se nanoparticles were found to exhibit higher solubility, greater bioactivity, and lower cytotoxicity compared to metal-based Se compounds like selenate and selenite^29,30^. Moreover, Se nanoparticles improved root formation and organogenesis in tobacco (*Nicotiana tabacum*) under controlled conditions, whereas selenate inhibited these processes^31^. Metal-based nanomaterials are non-degradable, highly reactive, and persistent in the food chain, which poses unexpected health risks^32^. Cs-NPs have been employed as carriers for pesticides, herbicides, fertilizers, and plant growth regulators. Cs-NPs protect biomolecules from damage caused by temperature, light, and pH fluctuations. Additionally, Cs-NPs encapsulated within a Cs matrix exhibit great potential in agriculture^33^. The application of chitosan-based nanoparticles, especially Cs-Pro at 400 mg L⁻¹, effectively enhanced wheat’s tolerance to salt stress through improvements in water retention, photosynthetic pigment stability, antioxidant activity, and ion homeostasis at the molecular level. These effects were genotype-dependent, with the salt-tolerant cultivar showing the most pronounced improvements. These findings underscore the potential of Cs-based nanoparticle formulations as a sustainable, genotype-sensitive solution for improving wheat performance under saline conditions^34^. Earlier research investigated the foliar application of Cs–Se NPs on bitter melon plants under varying salinity levels. The study found that a 20 mg/L concentration of Cs–Se NPs significantly improved plant height, chlorophyll content, and biomass under salt stress conditions^35^. Another study also demonstrated that green-synthesized Se nanoparticles significantly improved the growth of barley seedlings under salt stress. The application of Se NPs led to increased shoot dry weight and enhanced antioxidant enzyme activities, suggesting their role in mitigating oxidative damage caused by salinity^36^.

However, there are still many knowledge gaps in this area. On the one hand, the effects of NPs are even less known in barley than in many other plant species, despite the fact that barley is also one of the most important economic crops. On the other hand, the methods of use and the effectiveness of NPs depend on many factors that require further studies. The purposes of this study are to develop and validate an innovative, safe, and effective nano-enabled strategy using Se and chitosan nanoparticles to improve barley’s growth and productivity under salinity stress by enhancing its physiological and molecular defense systems. Furthermore, since knowledge about molecular mechanisms is also incomplete, further aim was to better understand the genotype-dependence and the mode of action of nano-treatments under control and saline conditions at biochemical and gene expression levels. This research could contribute to sustainable agriculture by enabling barley cultivation on saline soils and reducing yield losses caused by salinity.

## Materials and methods

### Plant material and growing conditions

The experiment was carried out in the research greenhouses of the HUN-REN Centre for Agricultural Research Hungary, Martonvásár, as a factorial experiment using a completely randomized design. Seeds of barley plants (*Hordeum vulgare* L., cv, Mv Initum, and Tectus) were placed in sodium hypochlorite solution (1%) for 5 min and then washed extensively with sterile distilled water. After 6 days, the germinated seeds were planted in pots (5 seeds per pot) of soil mixture and 25/20°C (day/night) temperatures. After 21 days, at the four leaf stage, plants were sprayed with Cs, Se, Cs-Se NPs and distilled water as a control for three days, then salt stress was applied for three days at concentrations of 0, 100, and 200 mM NaCl. The volume of applied saline solution to each pot was 200 mL, while the control group was supplied with 200 mL distilled water. Subsequently, the leaf samples were collected at day 28, instantly frozen in liquid N_2_ and kept for additional analysis at -80 °C.

### Preparation of chitosan–selenium NPs (Cs-Se NPs)

Cs with 75–85% deacetylation (310–375 kDa molecular weight, CAS Number: 9012-76-4), sodium selenite (CAS Number: 10102-18-8) and tripolyphosphate (TPP, CAS Number: 7758-29-4) were obtained from Sigma-Aldrich Co (St Louis, MO, USA). Deionized water (DI) was used for this experiment. Cs-NPs were prepared according to Jafari et al.^37^. In summary, a Cs solution was obtained by adding 1 g of Cs powder to 1 L of DI and 1000 µL of acetic acid under stirring for 1 h at room temperature. Separately, 0.1 g of sodium selenite was added to 100 mL of DI and dissolved by shaking vigorously. The selenite solution added to the Cs solution separately. The ratio of Cs to TPP by weight was 2.5:1, so 0.4 g of TPP was dissolved in 50 mL of DI and slowly added to the Cs-Se solutions.

### Plant growth parameters

At the end of the experiment, i.e. after three days of salt treatments, the height of plants was measured using a ruler. Then the plants were cut at soil level, and the whole shoots were weighed for fresh weight (FW). After this the dry weight (DW) was determined after 48 h drying at 70 °C.

### Proline, chlorophyll and carotenoid measurements

The PRO content was determined on the basis of its reaction with ninhydrin, according to the Bates method^38^. The chlorophyll content was first estimated using a SPAD chlorophyll meter (Konika Minolta Ltd., Osaka, Japan). Then pigments involved in photosynthesis, including chlorophyll a (Chl a), chlorophyll b (Chl b), total chlorophyll contents (Chl a + Chl b) and carotenoid contents (CAR) were measured using the Lichtenthaler method^39^. In brief, chlorophylls were extracted from 0.5 g of fresh leaf samples with 80% acetone, then centrifuged at 6000 rpm for 10 min and incubated in the dark at 25 °C for 5 min. The absorbance of the extracts was measured with a spectrophotometer at 470, 645, and 663 nm. Finally, chlorophyll and carotenoid contents were calculated in mg L^− 1^ using specific equations and converted to mg g^− 1^ FW.

$${\mathrm{Chlorophyll}}\,{{a}}\left( {{\mathrm{mg}}\,{\mathrm{L}}^{{ - {\mathrm{1}}}} } \right) = {\mathrm{12}}.{\mathrm{7}}\,{\mathrm{A}}_{{{\mathrm{663}}}} - {\mathrm{2}}.{\mathrm{69}}\,{\mathrm{A}}_{{{\mathrm{645}}}}$$

$${\mathrm{Chlorophyll}}\,b\left( {{\mathrm{mg}}\,{\mathrm{L}}^{{ - {\mathrm{1}}}} } \right) = {\mathrm{22}}.{\mathrm{9}}\,{\mathrm{A}}_{{{\mathrm{645}}}} - {\mathrm{4}}.{\mathrm{68}}\,{\mathrm{A}}_{{{\mathrm{663}}}}$$

$${\mathrm{Total}}\,{\mathrm{chlorophyll}}\left( {{\mathrm{mg}}\,{\mathrm{L}}^{{ - {\mathrm{1}}}} } \right) = {\mathrm{chlorophyll}}\,a + {\mathrm{chlorophyll}}\,b$$

$${\mathrm{Carotenoids}}\left( {{\mathrm{mg}}\,{\mathrm{L}}^{{ - {\mathrm{1}}}} } \right) = ({\mathrm{1}}000\,{\mathrm{A}}_{{{\mathrm{47}}0}} - {\mathrm{1}}.{\mathrm{82}}\,{\mathrm{chlorophyll}}\,a - {\mathrm{85}}.0{\mathrm{2}}\,{\mathrm{chlorophyll}}\,b)/{\mathrm{198}}$$

### Antioxidant enzymes activities

Measurements for antioxidant enzyme activities were performed as previously described in Pál et al.^40^. The CAT (EC 1.11.1.6) activity was measured spectrophotometrically by monitoring the decrease in absorbance at 240 nm, and APX (EC 1.11.1.11) activity was determined at 290 nm with a spectrophotometer (Shimadzu UV-VIS 160 A). Enzyme activities were expressed in nkatal g^− 1^ fresh weight (FW).

### Real-Time PCR

Total RNA was extracted from leaf tissues of three independent barley plants of each treatments using TRIzol reagent according to the manufacturer’s guidelines. RNA samples quantity was determined by NanoDrop (2000c, Thermo Scientific, USA). RNA samples were subjected for DNaseI treatment and removed with a Direct-zol ™ RNA MiniPrep Kit (Zymo Research, Irvine, CA, USA) according to the manufacturer’s instructions. RNA to cDNA was converted using M-MLV reverse transcriptase from (Promega Corporation, Madison, WI, USA). *Hvcyclo* was stable under different conditions, was used as a reference gene for RT-qPCR analysis. PCRBIO SyGreen Mix (PCR Biosystems, London, UK) and CFX96 Real-Time PCR Detection System (Bio-Rad, Hercules, CA, USA) were used for RT-qPCR. Three biological replicates and three technical replicates were performed for each treatment, and the relative expression of selected genes (Table 1) were compared by the 2^−ΔΔCT^ method^41^.

### Statistical analyses

The statistical analysis of data was performed based on the subsequent LSD’s range test at a significance level of *P* ≤ 0.05 with three replicates, using SAS software version 9.4^42^. Correlation analyses was assessed to determine the relationships between the traits using SRplot^43^. GraphPad Prism (version 9.0.1) was employed to create visual representations of the data associated with gene expression patterns.

**Table 1:** Genes and primers used for RT-qPCR analyses in the present study.

Gene	Forward primer (5’-3’)	Reverse primer (5’-3’)
*HvCyclo*	CCTGTCGTGTCGTCGGTCTAAA	ACGCAGATCCAGCAGCCTAAAG
*HvHKT2*	GACCCTTTCTCCACCGATTA	CACGAGCCGATTTACACG
*HvNHX1*	TGCATATCTACCAGTGCTTAT	GGTTCAAGACACAAGTTCAGT
*HvNHX2*	GGTTTTCGGCTTGCTGACTAA	CATTGGGCGCATGAACTTATC
*HvNHX3*	TGAGCCGAACATTACTGTGAT	ACGAGCTTACCTTTCAATACA
*HvSOS1*	GGCACCAACAGGAAGATGAA	GATATGCAGGAGGCCAGAGA
*HvSOS3*	GCTGCACCTCGAAAATCC	AAACCGCTCGTCACTGCT
*HvCAT2*	TGCAGGAGTACTGGCGTCTTCGACTT	AGATCCCGGGCACGAGGCCGGGGCC
*HvAPX*	GGAGTTGTCGCCGTGGAGGTGTCCGGTG	CAAGATCACCCTGGTCGCGCATAGTAGC
*HvSOD*	ATGGTGAAGGCTGTTGCTGTGC	TCAGCCTTGAAGTCCGATGATCCC

## Results and discussion

### Plant growth parameters

The ANOVA results indicated that salinity (S), cultivar (C), and nanoparticle treatments (Cs, Se, Cs–Se NPs) and their interactions significantly influence a wide range of morpho-physiological traits in barley under salt stress (Table 2). Notably, salinity adversely affected plant height, FW, DW, and pigment contents such as Chl a, Chl b, CAR and Chl a + Chl b, while nanoparticle applications significantly improved these traits, indicating their potential in enhancing salt stress tolerance. Apart from CAR, the cultivar effect was consistently significant, showing that genetic differences between Mv Initium and Tectus play a critical role in stress response. Interaction effects, especially between salinity and nanoparticles (S × NT) and cultivar and nanoparticles (C × NT), were significant for most traits, highlighting the importance of treatment combinations (Table 2). Overall, nanoparticle treatments, particularly when tailored to specific cultivars and salinity levels, show promise as a strategy to mitigate salt stress in barley.

**Table 2:** Analysis of variance the impact of foliar spraying of Cs, Se and Cs–Se NPs on morpho-physiological properties of two barley cultivars (Mv Initium, Tectus) under salt stress (0, 100 and 200 mM NaCl).

SOV		Mean squares							
	DF	Height	SPAD	FW	DW	Chl a	Chl b	Chl a + Chl b	CAR
S	2	**	ns	**	**	**	**	**	**
R	2	–	–	–	–	–	–	–	–
C	1	**	**	**	**	**	**	**	ns
NT	3	**	**	**	**	**	**	**	**
S × C	2	**	ns	**	**	**	**	**	*
S × NT	6	**	ns	**	**	**	**	**	*
C × NT	3	**	**	**	**	*	ns	*	ns
S × C × NT	6	**	ns	**	**	**	**	**	ns
Error	46	–	–	–	–	–	–	–	–

SOV = sources of variations; R = replicate; S = salinity; C = cultivar; NT = nanoparticle treatments; DF = degree of freedom; FW= fresh weight; DW = dry weight. Chl a = chlorophyll a; Chl b = chlorophyll b; Chl a + Chl b = total chlorophyll content; CAR = carotenoid; ns: not significant. *, **: significant at 1% and 5% probability level

The mean data clearly show that salinity stress negatively impacts barley growth traits, including plant height, FW, and DW, with increasing severity from 0 to 200 mM NaCl (Table 3). Across all salinity levels, Mv Initium generally performed better than Tectus, particularly in maintaining higher FW and DW. Application of nanoparticles Cs, and especially Cs–Se NPs, significantly mitigated the adverse effects of salt stress on both cultivars (Table 3) and showed the most consistent and substantial improvements in plant height, SPAD values, and biomass at all salinity levels, especially under 100 and 200 mM NaCl. Notably, Mv Initium treated with Cs–Se NPs in the control condition had the highest height (47.53 cm), suggesting a strong positive growth promoting effect. Under 100 and 200 mM NaCl, nanoparticle treatments significantly improved plant performance compared to controls, especially in, FW and DW, where Cs–Se treated plants consistently had higher values (Table 3). Overall Cs–Se NPs were the most effective across both cultivars and salinity levels, followed by Cs, while Se alone had limited effects. These findings highlight the potential of nanoparticle-based foliar treatments, particularly Cs–Se NPs, as a promising strategy to enhance salt stress tolerance in barley, with cultivar-specific responses emphasizing the importance of genotype in stress management.

**Table 3:** The effect of Cs, Se and Cs–Se NPs on morpho-physiological properties of two barley cultivars (Mv Initium, Tectus) under salt stress (0, 100 and 200 mM NaCl). In each column, values followed by the same letter(s) do not have significant difference at α = 0.05. Distilled water was used as a control.

Salt	Cultivars	Treatments	Height (cm)	SPAD	FW (g)	DW (g)
0	Mv Initium	Control	39.66 ^d^	29.70 ^ef^	0.76 ^d^	0.09 ^f^
		Cs	44.36 ^b^	34.70 ^ab^	0.86 ^b^	0.18 ^b^
		Se	42.66 ^c^	32.10 ^c^	0.79 ^c^	0.13 ^d^
		Cs–Se	47.53 ^a^	35.60 ^a^	0.91 ^a^	0.2 ^a^
	Tectus	Control	33.26 ^h^	30.34 ^de^	0.51 ^j^	0.03 ^l^
		Cs	43.93 ^cd^	31.74 ^cd^	0.58 ^h^	0.07 ^h^
		Se	34.93 ^gh^	29.42 ^ef^	0.54 ^i^	0.05 ^k^
		Cs–Se	44.36 ^b^	35.93 ^a^	0.67 ^f^	0.06 ^i^
100 mM	Mv Initium	Control	31.66 ^ij^	30.43 ^de^	0.54 ^i^	0.06 ^i^
		Cs	38.93 ^de^	31.95 ^cd^	0.70 ^e^	0.10 ^e^
		Se	34.66 ^gh^	29.65 ^ef^	0.65 ^fg^	0.07 ^h^
		Cs–Se	37 ^ef^	32.70 ^c^	0.79 ^c^	0.14 ^c^
	Tectus	Control	31.90 ^ij^	28.16 ^f^	0.43 ^kl^	0.02 ^m^
		Cs	33.33 ^h^	31.00 ^cd^	0.54 ^i^	0.05 ^k^
		Se	32.60 ^i^	30.65 ^de^	0.45 ^k^	0.03 ^l^
		Cs–Se	39.93 ^d^	32.40 ^c^	0.53 ^ij^	0.05 ^k^
	Mv Initium	Control	28.73 ^kl^	26.34 ^g^	0.34 ^mn^	0.02 ^m^
		Cs	31.36 ^ij^	31.49 ^cd^	0.62 ^g^	0.08 ^g^
200 mM		Se	29.20 ^jk^	27.64 ^fg^	0.36 ^m^	0.04 ^kl^
		Cs–Se	35.63 ^fg^	30.56 ^de^	0.60 ^gh^	0.12 ^d^
	Tectus	Control	28.33 ^kl^	24.34 ^h^	0.31 ^n^	0.02 ^m^
		Cs	31.13 ^ij^	30.56 ^de^	0.45 ^k^	0.03 ^l^
		Se	30.83 ^jk^	26.42 ^g^	0.37 ^m^	0.02 ^m^
		Cs–Se	31.30 ^ij^	32.16 ^c^	0.43 ^kl^	0.04 ^kl^

FW= fresh weight; DW = dry weight.

### Photosynthetic pigments

**Fig. 1:**
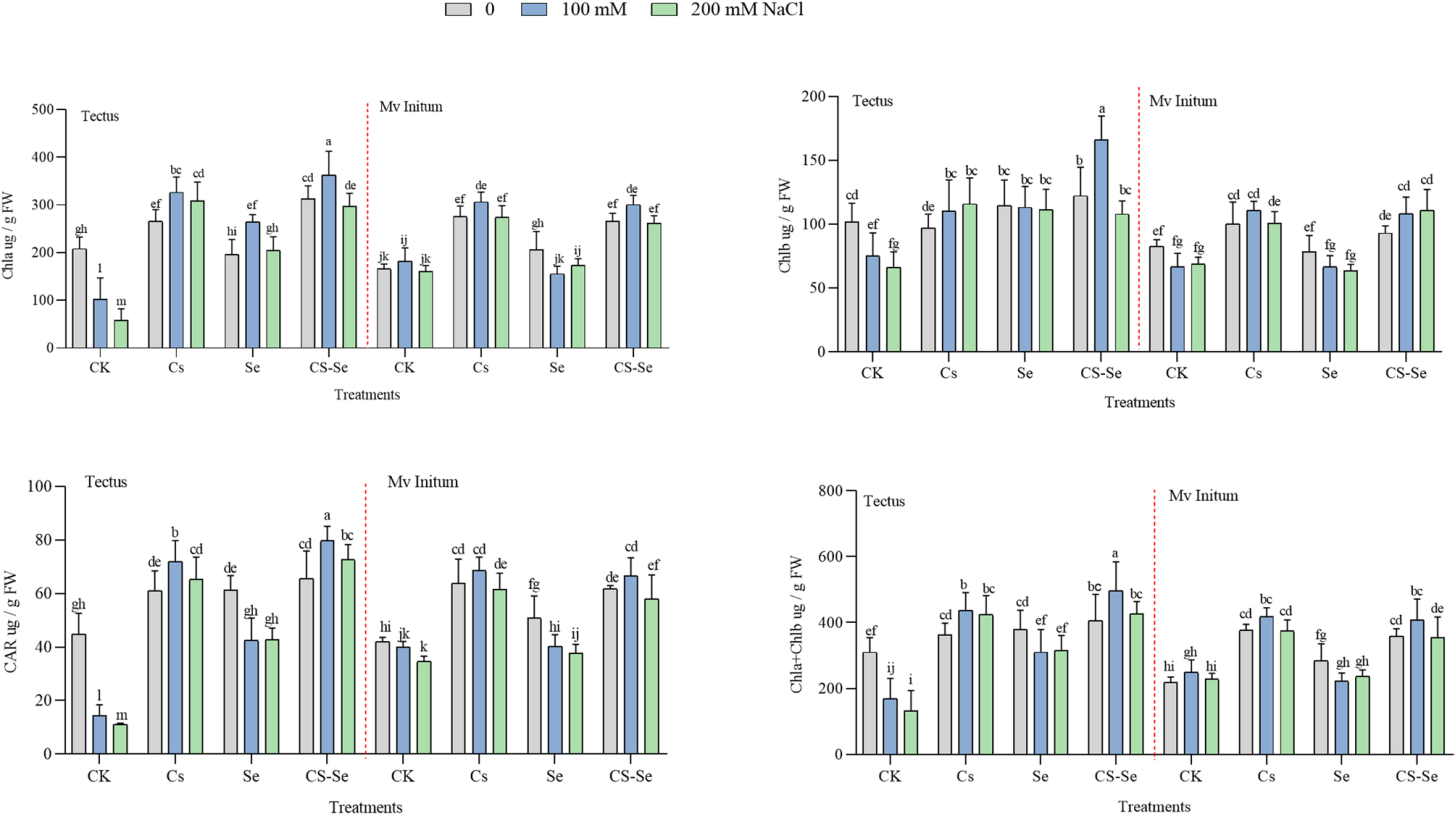
The effect of Cs, Se and Cs–Se NPs on Chl a = chlorophyll a; Chl b = chlorophyll b; Chl a + Chl b = total chlorophyll content and CAR = carotenoid of two barley cultivars (Mv Initium, Tectus) under salt stress (0, 100 and 200 mM NaCl).

Similarly, to the biomass parameters, SPAD values were also the highest in the Cs-Se and Cs treated plants, although, the effect of salinity was less pronounced in this parameter (Table 2). However, increasing NaCl concentration generally reduced Chl a content in barley leaves (Fig. 1) but foliar application of NPs, especially Cs–Se NPs mitigates this reduction, especially at higher salt concentrations, leading to significant improvements in Chl a compared to untreated plants. Similarly to Chl a, Chl b content decreases with increasing salt stress. Cs–Se NPs help maintain or increase Chl b levels under salt stress, though the effect may be less pronounced than for Chl a. Chl a + Chl b declines under salt stress but is significantly higher in Cs–Se NPs treated plants, especially under saline conditions. CAR content, which is important for plant stress tolerance, also drops under salt stress (Fig. 1). Application of Cs–Se NPs boosts CAR levels under these conditions, helping plants better cope with oxidative stress. The enhancement in pigment retention is attributed to the protective role of Se in mitigating oxidative stress and sustaining chloroplast integrity^44,45^. Present data are in accordance with earlier results that nano-Se and related nanoparticle treatments can improve chlorophyll and carotenoid contents, thus enhancing plant resilience to salinity^35,46–48^. These findings align with previous reports that nanoparticle treatments enhance plant stress resilience via enhancement of plant growth, photosynthetic efficiency^49,50^. However, the genotype-dependent mode of action of the Cs-Se NPs in barley plants is still unknown.

### Proline accumulation

**Fig. 2:**
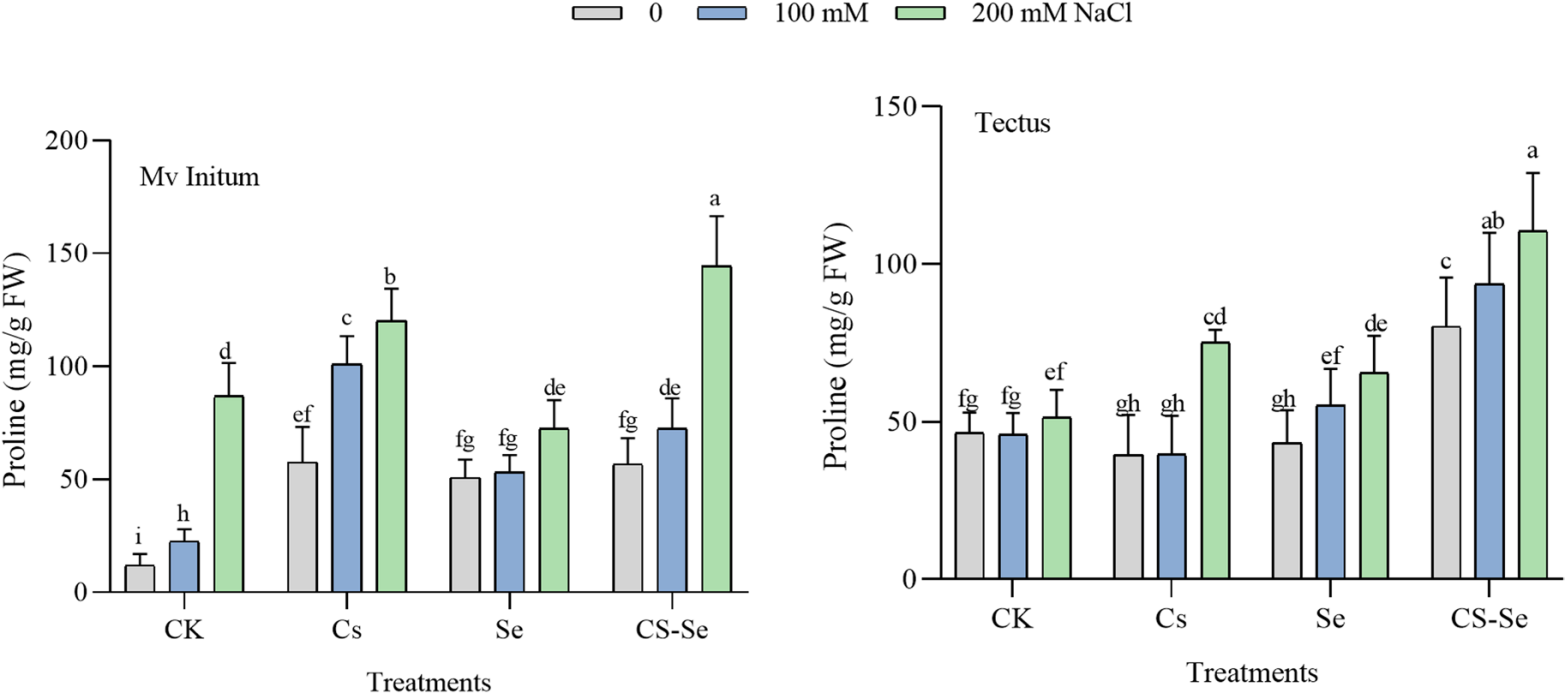
The effect of Cs, Se and Cs–Se NPs on PRO of two barley cultivars (Mv Initium, Tectus) under salt stress (0, 100 and 200 mM NaCl).

In order to better understand the molecular and physiological mechanisms behind the effects of Cs-Se foliar spray, first the level of PRO was determined. PRO accumulation is a common biochemical response to osmotic stress, helping to stabilize proteins and membranes^51^. Under the present experimental conditions, it increased significantly under salt stress (100 and 200 mM NaCl) in both barley cultivars Mv Initium and Tectus. Treatments with Cs, Se, and especially Cs–Se NPs further enhanced PRO accumulation compared to the control (distilled water). Cs–Se NPs showed the strongest effect, with the highest PRO content observed at 200 mM NaCl (Fig. 2). The increase in PRO content in Cs-Se treated plants suggests an improved osmotic adjustment mechanism, facilitating stress tolerance^52^.

**Table 3:** The effect of Cs, Se and Cs–Se NPs on morpho-physiological properties of two barley cultivars (Mv Initium, Tectus) under salt stress (0, 100 and 200 mM NaCl). In each column, values followed by the same letter(s) do not have significant difference at α = 0.05.

Salt	Cultivars	Treatments	Height (cm)	SPAD	FW (g)	DW (g)
0	Mv Initium	Control	39.66 ^d^	29.70 ^ef^	0.76 ^d^	0.09 ^f^
		Cs	44.36 ^b^	34.70 ^ab^	0.86 ^b^	0.18 ^b^
		Se	42.66 ^c^	32.10 ^c^	0.79 ^c^	0.13 ^d^
		Cs–Se	47.53 ^a^	35.60 ^a^	0.91 ^a^	0.2 ^a^
	Tectus	Control	33.26 ^h^	30.34 ^de^	0.51 ^j^	0.03 ^l^
		Cs	43.93 ^cd^	31.74 ^cd^	0.58 ^h^	0.07 ^h^
		Se	34.93 ^gh^	29.42 ^ef^	0.54 ^i^	0.05 ^k^
		Cs–Se	44.36 ^b^	35.93 ^a^	0.67 ^f^	0.06 ^i^
100 mM	Mv Initium	Control	31.66 ^ij^	30.43 ^de^	0.54 ^i^	0.06 ^i^
		Cs	38.93 ^de^	31.95 ^cd^	0.70 ^e^	0.10 ^e^
		Se	34.66 ^gh^	29.65 ^ef^	0.65 ^fg^	0.07 ^h^
		Cs–Se	37 ^ef^	32.70 ^c^	0.79 ^c^	0.14 ^c^
	Tectus	Control	31.90 ^ij^	28.16 ^f^	0.43 ^kl^	0.02 ^m^
		Cs	33.33 ^h^	31.00 ^cd^	0.54 ^i^	0.05 ^k^
		Se	32.60 ^i^	30.65 ^de^	0.45 ^k^	0.03 ^l^
		Cs–Se	39.93 ^d^	32.40 ^c^	0.53 ^ij^	0.05 ^k^
	Mv Initium	Control	28.73 ^kl^	26.34 ^g^	0.34 ^mn^	0.02 ^m^
		Cs	31.36 ^ij^	31.49 ^cd^	0.62 ^g^	0.08 ^g^
200 mM		Se	29.20 ^jk^	27.64 ^fg^	0.36 ^m^	0.04 ^kl^
		Cs–Se	35.63 ^fg^	30.56 ^de^	0.60 ^gh^	0.12 ^d^
	Tectus	Control	28.33 ^kl^	24.34 ^h^	0.31 ^n^	0.02 ^m^
		Cs	31.13 ^ij^	30.56 ^de^	0.45 ^k^	0.03 ^l^
		Se	30.83 ^jk^	26.42 ^g^	0.37 ^m^	0.02 ^m^
		Cs–Se	31.30 ^ij^	32.16 ^c^	0.43 ^kl^	0.04 ^kl^

FW= fresh weight; DW = dry weight.

The increase in PRO under salt stress aligns with its role as an osmoprotectant and ROS scavenger, helping maintain cellular osmotic balance and protecting proteins under stress.

The further enhancement by Cs–Se NPs suggests that these nanoparticles stimulate osmolyte synthesis, improving stress tolerance more effectively than Se alone, consistent with findings that Se and Se-based nanomaterials boost PRO levels in salt-stressed plants^35,53^. Positive correlations were observed between growth parameters FW, DW and photosynthetic pigments (Chl a, Chl b, Chl a + Chl b, and CAR). This indicates that higher pigment content is associated with better growth performance under salt stress (Fig. 4). PRO, a known osmoprotectant, showed significant positive correlations with antioxidant enzymes APX and CAT, suggesting that PRO accumulation is linked with enhanced antioxidant defense under salinity (Fig. 4). The positive correlations between photosynthetic pigments and growth traits suggest that maintenance of chlorophyll and carotenoid content under salt stress supports better growth and yield performance, consistent with findings that salt stress decreases these pigments but tolerant genotypes maintain higher levels^54^.

### Antioxidant enzymes activities

**Fig. 3:**
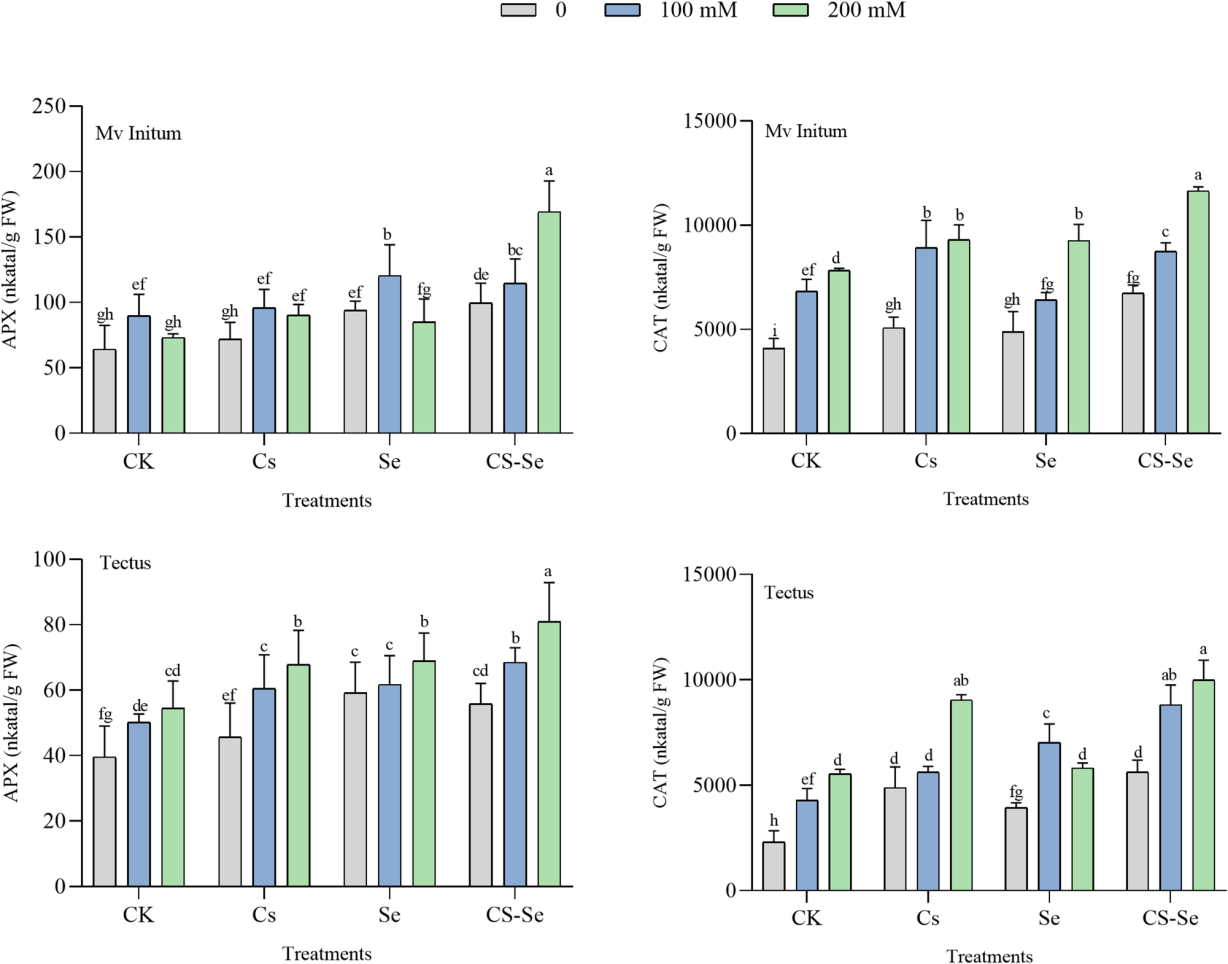
The effect of Cs, Se and Cs–Se NPs on APX and CAT enzyme activities of two barley cultivars (Mv Initium, Tectus) under salt stress (0, 100 and 200 mM NaCl).

**Fig. 4:**
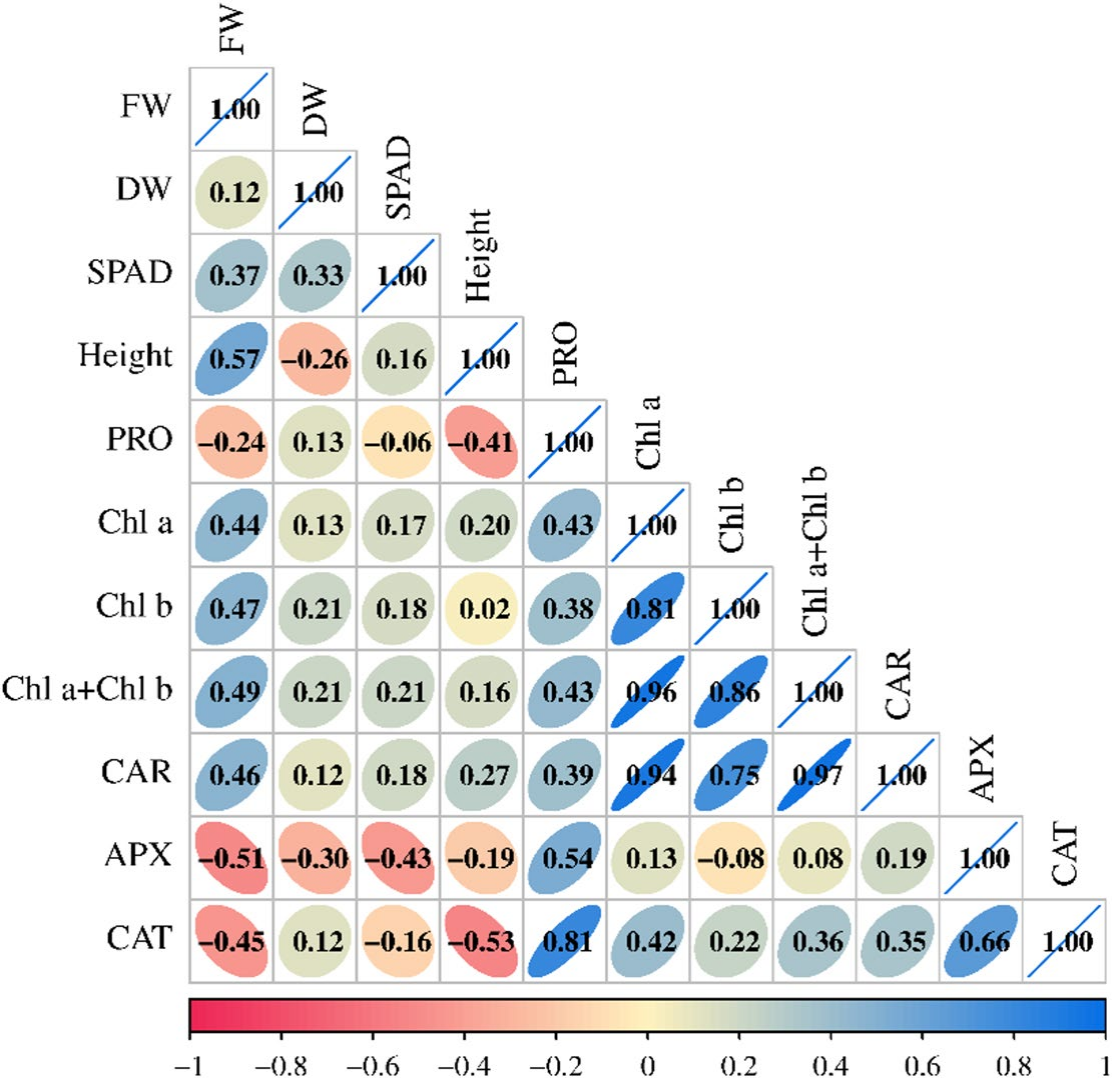
Pearson correlation coefficients of morphological properties two barley cultivars (Mv Initium and Tectus) under salt stress (0, 100 and 200 mM NaCl). FW= fresh weight; DW = dry weight; PRO = proline; Chl a = chlorophyll a; Chl b = chlorophyll b; Chl a + Chl b = total chlorophyll content; CAR = carotenoid; APX = ascorbate peroxidase; CAT = catalase;

Salt stress induces oxidative stress via ROS, which plants counteract by upregulating antioxidant enzymes like APX and CAT. Besides its role as an osmoregulator, proline may also serve as a stress signal molecule, influencing certain gene expression and metabolic processes. Its positive correlation with antioxidant enzymes indicates a linked protective mechanism against oxidative damage induced by salt stress^54,55^. Activities of APX and CAT increased under salt stress in both cultivars, which supports improved ROS scavenging capacity. Although direct measurements of ROS content were not reported, the significant upregulation of antioxidant enzyme genes (*HvAPX*, *HvSOD*, *HvCAT*) following Cs-Se NPs treatment under salinity strongly indicates enhanced ROS detoxification. The coordinated increase in antioxidant enzymes helps scavenge ROS generated under salt stress, reducing lipid peroxidation and membrane damage. This is supported by observations of lower malondialdehyde content and better membrane stability index in tolerant genotypes^55^. Application of Cs–Se NPs further elevated APX and CAT activities compared to untreated controls. Cs–Se NPs treatment led to the most pronounced increase in enzyme activities in 200 mM NaCl, indicating enhanced antioxidant defense (Fig. 3). The elevated enzyme activities with Cs–Se NPs treatment indicate that these nanoparticles enhance the plant’s antioxidative capacity, reducing oxidative damage. This supports previous reports that Se and Cs–Se NPs application improves antioxidant enzyme activities and mitigates salt-induced oxidative stress^35,53^. APX and CAT were positively correlated with each other, reflecting coordinated upregulation of antioxidant defense mechanisms in response to salt stress (Fig. 4).

### Gene expression

Increasing salt concentrations (100 and 200 mM NaCl) generally altered gene expression levels compared to the non-saline control (0 mM NaCl), indicating that salt stress significantly impacts gene regulation in barley. In another study, a salt-tolerant barley Tunisian landrace (Boulifa) exposed to 200 mM NaCl, thousands of genes were differentially expressed in especially in the roots, but also in the leaves, after 2, 8, and 24 h of salt stress.

**Fig. 5:**
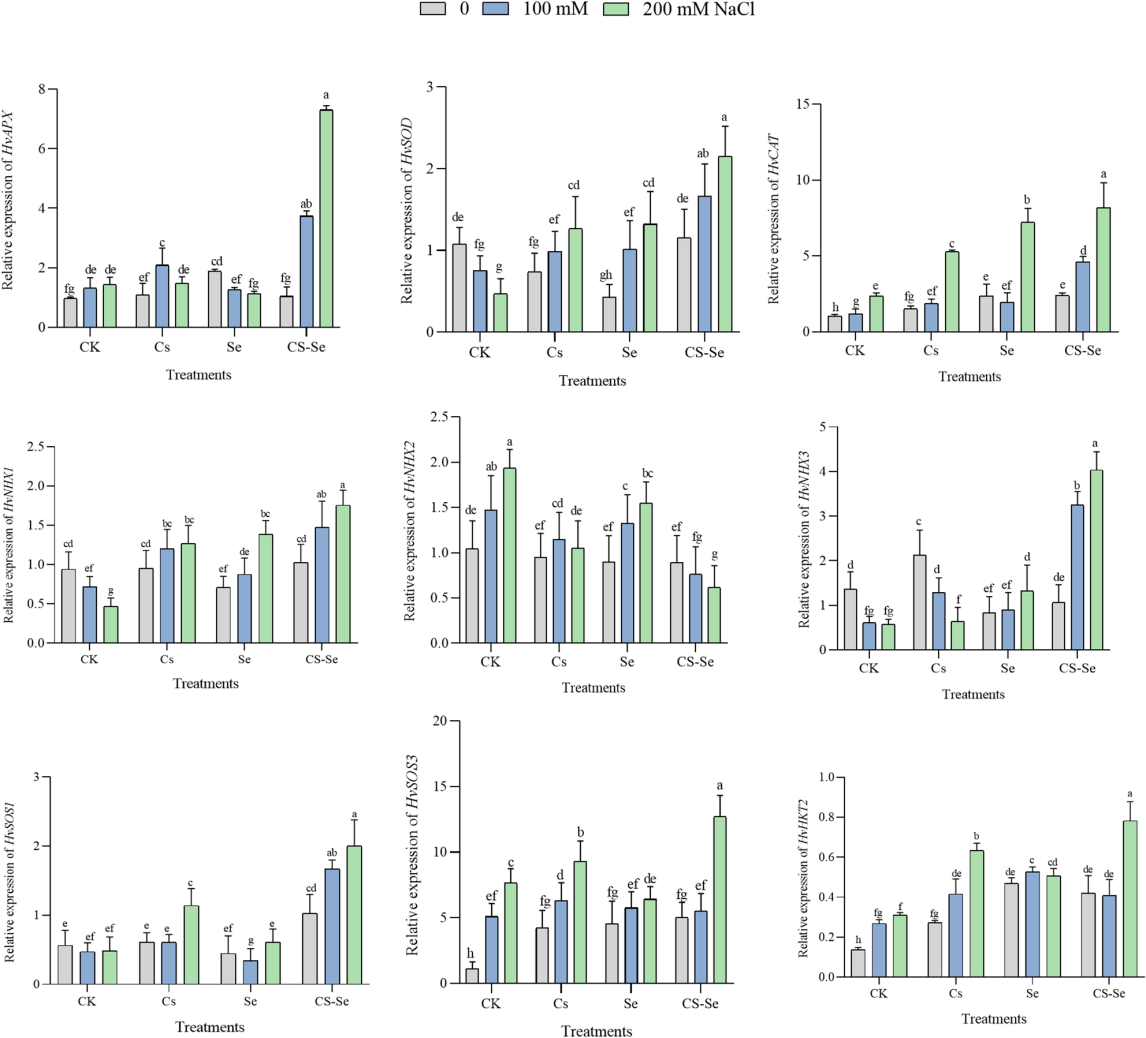
The effect of Cs, Se and Cs–Se NPs on the expression patterns of barley cultivar (Mv Initium) under salt stress (0, 100 and 200 mM NaCl). Each column is the mean expression of three technical and biological replicates. Error bars are standard deviations of biological replicates. Lower-case letters above bars indicate mean comparisons from LSD test at *p <* 0.05.

**Fig. 6:**
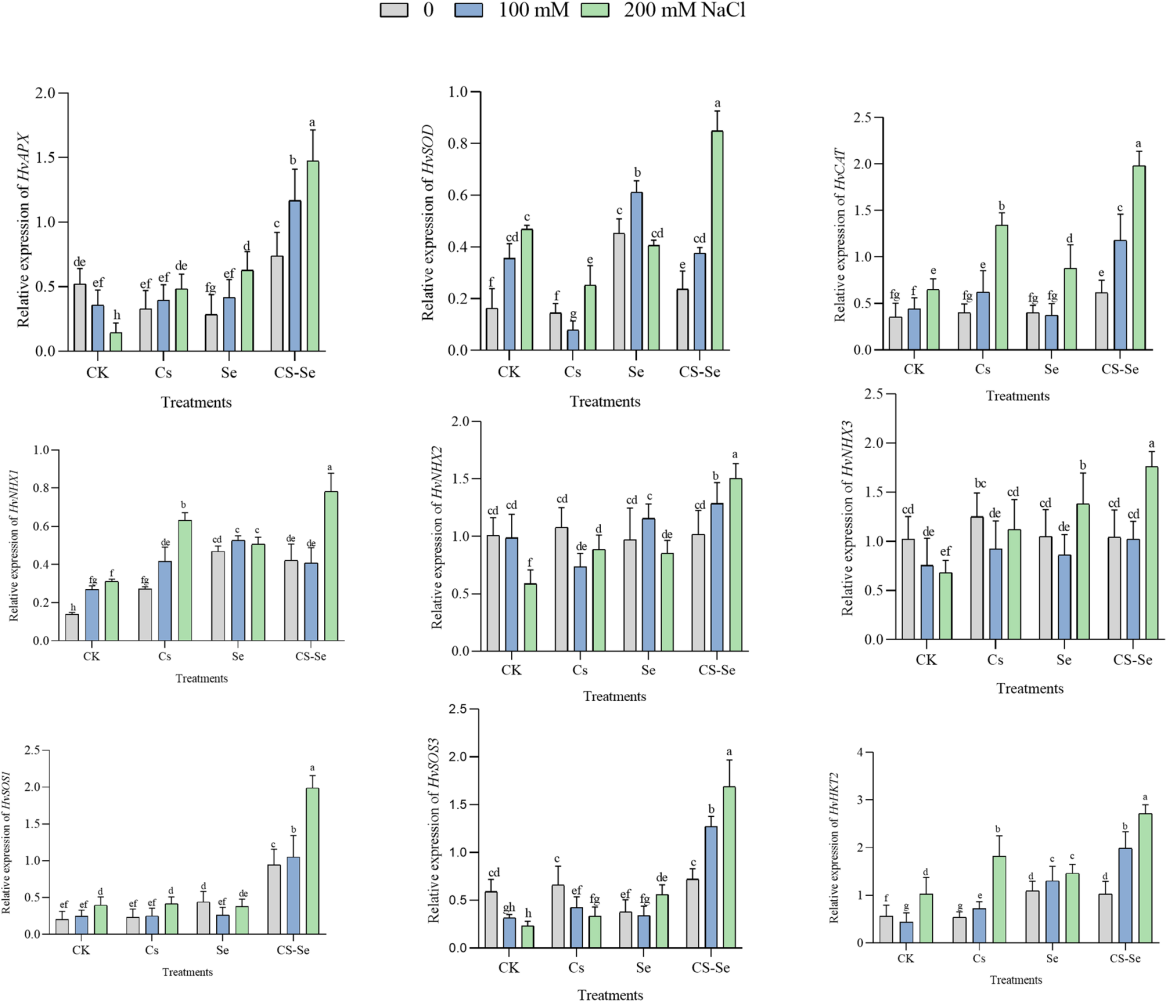
The effect of Cs, Se and Cs–Se NPs on the expression patterns of barley cultivar (Tectus) under salt stress (0, 100 and 200 mM NaCl). Each column is the mean expression of three technical and biological replicates. Error bars are standard deviations of biological replicates. Lower-case letters above bars indicate mean comparisons from LSD test at *p <* 0.05.

The highest number of differentially expressed genes (DEGs) occurred around 8 h of salt exposure, indicating a dynamic and time-dependent response^56^. CAT and APX genes are part of the antioxidant defense system activated under salt stress in barley and wheat. Their upregulation helps scavenge ROS generated by salt-induced oxidative stress, protecting cellular components and improving stress tolerance^34^. In the present work, under control conditions, the behavior of the investigated genes encoding antioxidant enzymes were similar as it was observed earlier^56^. However, the NPs treatment could modify the responses of certain genes to high salinity. For example, while in control Mv Initium plants, increasing salinity led to decreasing *HvSOD* expression level, in NPs treated plants, an increasing trend could be observed. The *HvAPX*,* HvSOD and HvCAT* genes encoding APX, SOD and CAT, respectively, exhibited the highest expression levels in both genotypes under Cs-Se treatment at 200 mM NaCl in the leaves (Figs. 5 and 6), reinforcing the role of nanoparticles in scavenging ROS and enhancing cellular defense mechanisms^22,30^. In agreement with another earlier works^57^, salinity stress substantially upregulated the mean relative expression of key barley genes involved in salt tolerance, including *HvSOS1*, *HvSOS3*, *HvHKT2*, *HvNHX1*, and *HvNHX3*, compared to control conditions, highlighting their crucial role in maintaining ion homeostasis via Na^+^ exclusion and sequestration under stress. Under the present experimental conditions, significant increase was also found in the expression level of *HvNHX2* in Mv Initium, but not in Tectus (Figs. 5 and 6).

However, the effects of salinity were significantly influenced by NP treatments in addition to genotype. Previous studies indicating that Se nanoparticles modulate ionic balance and mitigate Na^+^ toxicity^58,59^. In the present study, treatment with Cs–Se NPs usually modulated gene expression more effectively than Se alone under salt stress conditions. The expression analysis of key ion homeostasis-related and antioxidant genes (*HvAPX*,* HvSOD*,* HvCAT*,* HvNHX1*,* HvSOS1*,* HvSOS3*,* HvHKT2*) revealed significant upregulation in response to Cs-Se NPs treatment in the selected barley genotypes under salt stress (Figs. 5 and 6). At 100 and 200 mM NaCl, Cs–Se NPs treatment showed a statistically significant difference in expression levels compared to untreated controls and single-element treatments (*p* < 0.05). This suggests that Cs–Se NPs have a synergistic effect in enhancing salt stress tolerance at the molecular level (Fig. 5).

Earlier studies have also demonstrated that under moderate or severe saline conditions, salt-tolerant genotypes usually show upregulation of protective genes such as *HvNHX1* in roots and leaves, enhancing Na^+^ compartmentalization and tolerance, whereas sensitive genotypes often display downregulation^60^. Genetic variation in *HvNHX* alleles may correlate with salinity tolerance in wild and cultivated barley varieties^57,60^. These genes regulate ion homeostasis by sequestering Na^+^ into vacuoles, contributing to salt tolerance. Salt-sensitive genotypes show downregulation or less induction of these genes. In the present experiment, the behavior of *HvNHX1* differed between the genotypes: salinity decreased it in Mv Initium, but increased it in Tectus. However, their response was similar to NPs treatment. In contrast to this, after treatment with Cs-Se, *HvNHX2* increased in Mv Initium but decreased in Tectus under high level (200 mM) saline treatment. The behaviour of *HvNHX3* was almost the opposite: substantial increase in Mv Initum, but moderate changes in Tectus (Figs. 5 and 6).

In barley, *HvSOS1* and *HvSOS3* genes are significantly induced by salt stress, playing crucial roles in maintaining ion homeostasis and salt tolerance^57^. High-affinity K^+^ Transporter (*HKT*) genes mediate Na^+^ transport or Na^+^-K^+^ co-transport and play a critical role in salt tolerance by controlling Na^+^ uptake and distribution within the plant, thus maintaining ion homeostasis^61^. Genome-wide association studies (GWAS) in barley have mapped *HKT1*,5 to chromosome 4, linking SNP markers near this gene to salt tolerance traits. Expression analyses show tissue-specific regulation of *HKT1*,5 under salt stress, which is crucial for controlling Na+ distribution and minimizing toxicity in sensitive tissues^62^.

The observed gene expression pattern in the Tectus cultivar under 200 mM NaCl stress reveals a complex response indicative of its sensitivity to salinity. Specifically, the expression levels of key genes involved in stress tolerance (*HvNHX2*,* HvNHX3*, *HvSOS3*, and *HvAPX*) decreased under control treatment conditions, while other genes such as *HvSOS1*, *HvNHX1*, *HvHKT2*, *HvSOD*, and *HvCAT* showed increased expression. However, the magnitude of this increase was notably lower compared to both the Cs–Se NPs treatment and the Mv Initium cultivar, which is known for its greater stress tolerance (Fig. 6). Specifically, *HvSOS3* is part of the Salt Overly Sensitive (SOS) pathway critical for maintaining ion balance under salt stress, while *HvAPX* encode antioxidant enzyme that mitigate oxidative damage caused by reactive oxygen species during abiotic stress^63^. The relatively lower upregulation of these genes in Tectus indicates a weaker molecular defense response, which correlates with increased sensitivity to stress. This pattern is consistent with findings in barley and other cereals, where tolerant cultivars show stronger induction of key stress-responsive genes, including those involved in ion homeostasis and antioxidant defense, compared to sensitive cultivars (Fig. 6). For example, studies on heat and salt stress have demonstrated that tolerant genotypes maintain higher expression levels of ion transporters and antioxidant enzymes, correlating with better physiological performance and lower cellular damage, while sensitive genotypes have reduced or delayed gene activation and suffer from increased electrolyte leakage and oxidative stress^64^. The upregulation of key barley genes involved in salt tolerance (*HvSOS1*, *HvSOS3*, *HvHKT2*, *HvNHX1*, *HvNHX2* and *HvNHX3*) under salinity stress suggests that these genes play crucial roles in maintaining ion homeostasis, mainly by excluding Na^+^ from the cytoplasm and sequestering Na^+^ in vacuoles. The specific increase in *HvNHX2* expression in the Mv Initium barley cultivar indicates that this gene may have a role in Na^+^ compartmentalization and maintaining potassium ion balance under salt stress conditions in this cultivar, whereas it does not increase in the Tectus cultivar. Nanomaterials are known to influence gene expression related to ion transport and homeostasis, which can affect Na^+^ and K^+^ content in plants. By modulating the expression of genes like *HvNHX2*, nanomaterials potentially enhance the sequestration of Na^+^ into vacuoles and improve K^+^ retention or uptake, thereby preserving cellular ion balance under salinity stress. This effect contributes to improved salt tolerance by preventing toxic Na^+^ accumulation and sustaining essential K^+^ levels.

Supporting transcriptomic studies in barley have demonstrated that stress-tolerant cultivars exhibit a more robust and extensive gene expression reprogramming under drought, heat, and combined stresses compared to sensitive cultivars, which show fewer or less pronounced changes in expression of stress-related genes. This differential gene expression underpins physiological resilience and stress tolerance^65^. Thus, the comparative gene expression data between Tectus and Mv Initium aligns with established molecular evidence that the magnitude of upregulation of stress-responsive genes is a key determinant of cultivar sensitivity or tolerance to abiotic stress.

## Conclusion

This study confirmed that salinity stress significantly reduces barley growth, biomass, and photosynthetic pigments, with more severe effects at higher salt concentrations. Among the tested treatments, Cs-Se NPs showed the greatest potential to alleviate salt stress by markedly improving morpho-physiological traits including plant height, FW, DW, and chlorophyll content across both barley cultivars. The application of Cs-Se NPs enhanced antioxidant enzyme activities and PRO content, which are essential for osmotic adjustment and ROS scavenging under salt stress. Additionally, improved retention of photosynthetic pigments and increased PRO levels suggest stronger cellular protection against oxidative damage. Positive correlations between PRO accumulation and antioxidant enzyme activities further indicate a coordinated enhancement of ROS homeostasis mediated by Cs-Se NPs. Molecular analyses revealed that Cs-Se NPs significantly upregulated the expression of salt-responsive genes involved in antioxidant defense and ion homeostasis, highlighting their role in activating key molecular pathways that contribute to stress tolerance. These findings underscore the superior efficacy of Cs-Se NPs compared to individual Cs or Se treatments, demonstrating a synergistic and genotype-dependent effect. This nano-enabled approach offers a sustainable and innovative strategy to improve barley productivity on saline soils, potentially reducing yield losses due to salinity and supporting food security.

## Data Availability

All the data in the present study are included in this manuscript.

## Abbreviations

Cs: Chitosan
Se: Selenium
Cs-Se NPs: Chitosan-selenium nanoparticles
PRO: Proline
APX: Ascorbate peroxidase
CAT: Catalase
ROS: Reactive oxygen species
PAL: Phenylalanine ammonia lyase
TPP: Tripolyphosphate
HKT: High-affinity K^+^ transporter
SOS: Salt overly sensitive
CAR: Carotenoid contents

## References

[1] Giraldo, P., Benavente, E., Manzano-Agugliaro, F. & Gimenez, E. Worldwide research trends on wheat and barley: A bibliometric comparative analysis. *Agronomy* **9** (7), 352 (2019).

[2] Tricase, C., Amicarelli, V., Lamonaca, E. & RanaRL Economic analysis of the barley market and related uses. *Grasses Food Feed*. **10**, 25–46 (2018).

[3] Zhou, M. X. Barley production and consumption. In *Genetics and improvement of barley malt quality*. (Springer, 2009).

[4] Dawson, I. K. R. J. et al. A translational model for adaptation to climate change. New Phytol. **206**, 913–931 (2015). 10.1111/nph.13266 25605349

[5] Isayenkov, S. V. M. & FJM Plant salinity stress. Many unanswered questions remain. *Front. Plant. Sci.* **10**, 80–91 (2019). 30828339 10.3389/fpls.2019.00080 PMC6384275

[6] Sheteiwy, M. S. et al. The effects of microbial fertilizers application on growth, yield and some biochemical changes in the leaves and seeds of guar (*Cyamopsis tetragonoloba* L.). Food Res Int. **172**:113122 (2023). 10.1016/j.foodres.2023.113122 37689887

[7] Basit, F. et al. Deciphering the role of glycine betaine in enhancing plant performance and defense mechanisms against environmental stresses. Front Plant Sci. **16**, 1582332 (2025). 10.3389/fpls.2025.1582332 PMC12325374 40772049

[8] El-Ramady, H. et al. Review of crop response to soil salinity stress: Possible approaches from leaching to nano-management. *Soil. Syst.* **8** (1), 11 (2024).

[9] Hassani, A., Azapagic, A. & Shokri, N. Global predictions of primary soil salinization under changing climate in the 21st century. *Nat. Commun.* **12** (1), 6663 (2021). 34795219 10.1038/s41467-021-26907-3 PMC8602669

[10] Hualpa-Ramirez, E. et al. Stress salinity in plants: New strategies to cope with in the foreseeable scenario. Plant Physiol Biochem. 108507 (2024). 10.1016/j.plaphy.2024.108507 38467083

[11] Zhu, J. K. Plant salt tolerance. Trends Plant Sci. **6**:66–71 (2001). 10.1016/s1360-1385(00)01838-0 11173290

[12] Golldack, D. L., Mohan, C. & Probst, H. N. Tolerance to drought and salt stress in plants: Unraveling the signaling networks. Front Plant Sci. **5**:151–161 (2014). 10.3389/fpls.2014.00151 PMC4001066 24795738

[13] Schulte, D. C. et al. RP. The international barley sequencing consortium–At the threshold of efficient access to the barley genome. Plant Physiol. **149**:142–147 (2009). 10.1104/pp.108.128967 PMC2613708 19126706

[14] Irik, H. A. & Bikmaz, G. Effect of different salinity on seed germination, growth parameters and biochemical contents of pumpkin (*Cucurbita pepo* L.) seeds cultivars. Sci Rep. **14**(1):6929 (2024). 10.1038/s41598-024-55325-w PMC10960046 38519514

[15] Jogaiah, S. G. & Tran, S. R. LSP. Systems biology-based approaches toward understanding drought tolerance in food crops. Crit Rev Biotechnol. **33**:23–39 (2013). 10.3109/07388551.2012.659174 22364373

[16] Dai, F. N. et al. Tibet is one of the centers of domestication of cultivated barley. Proc Natl Acad Sci USA. **109**:16969–16973 (2012). 10.1073/pnas.1215265109 PMC3479512 23033493

[17] Yahiaoui S.C-M. *et al*. Spanish barley landraces outperform modern cultivars at low-productivity sites. *Plant. Breed.* **133**, 218–226 (2014).

[18] Iqbal, M. et al. Exogenously applied selenium reduces oxidative stress and induces heat tolerance in spring wheat. *Plant. Physiol. Biochem.* **94**, 95–103 (2015). 26057700 10.1016/j.plaphy.2015.05.012

[19] Moore, L. & Mahmoudkhani, A. Methods for removing selenium from aqueous systems (2011).

[20] Schwarz, K. & Foltz, C. M. Selenium as an integral part of factor 3 against dietary necrotic liver degeneration. *J. Am. Chem. Soc.* **79** (12), 3292–3293 (1957). 10408880

[21] Fordyce, F. M. Selenium deficiency and toxicity in the environment. In *Essentials of medical geology*. 2012:375–416.

[22] Kong, L., Wang, M. & Bi, D. Selenium modulates the activities of antioxidant enzymes, osmotic homeostasis and promotes the growth of sorrel seedlings under salt stress. *Plant Growth Regul*. **45**, 155–163 (2005).

[23] Diao, M. et al. Selenium promotes the growth and photosynthesis of tomato seedlings under salt stress by enhancing chloroplast antioxidant defense system. *Plant Growth Regul*. **33**, 671–682 (2014).

[24] Hawrylak-Nowak, B. Beneficial effects of exogenous selenium in cucumber seedlings subjected to salt stress. *Biol. Trace Elem. Res.* **132**, 259–269 (2009). 19434374 10.1007/s12011-009-8402-1

[25] Handa, N. et al. Selenium modulates dynamics of antioxidative defence expression, photosynthetic attributes and secondary metabolites to mitigate chromium toxicity in *Brassica juncea* L. *Environ. Exp. Bot.* **161**, 180–192 (2019).

[26] Duhan J. S. *et al*. Nanotechnology: The new perspective in precision agriculture. *Biotechnol. Rep.* **15**, 11–23 (2017). 10.1016/j.btre.2017.03.002 PMC5454086 28603692

[27] Gohari, G. et al. Titanium dioxide nanoparticles (TiO_2_ NPs) promote growth and ameliorate salinity stress effects on essential oil profile and biochemical attributes of *Dracocephalum moldavica*. *Sci Rep*. **10**:912 (2020). 10.1038/s41598-020-57794-1 PMC6976586 31969653

[28] Hussein, H. A. A., Darwesh, O. M. & Mekki, B. B. Environmentally friendly nano-selenium to improve antioxidant system and growth of groundnut cultivars under sandy soil conditions. *Biocatal. Agric. Biotechnol.* **18**, 101080 (2019).

[29] Djanaguiraman, M., Belliraj, N., Bossmann, S. H. & Prasad, P. V. High-temperature stress alleviation by selenium nanoparticle treatment in grain sorghum. ACS Omega. **3**(3), 2479–2491 (2018). 10.1021/acsomega.7b01934 PMC6641442 31458542

[30] Morales-Espinoza, M. C. et al. Se nanoparticles induce changes in the growth, antioxidant responses, and fruit quality of tomato developed under NaCl stress. *Molecules* **24** (17), 3030 (2019). 31438533 10.3390/molecules24173030 PMC6749263

[31] Domokos-Szabolcsy, E. et al. Accumulation of red elemental selenium nanoparticles and their biological effects in Nicotinia tabacum. *Plant. Growth Regul.* **68**, 525–531 (2012).

[32] Malerba, M. & Cerana, R. Recent applications of chitin-and chitosan-based polymers in plants. *Polymers* **11**, 839 (2019). 31072059 10.3390/polym11050839 PMC6572233

[33] Mujtaba, M. et al. Chitosan-based delivery systems for plants: A brief overview of recent advances and future directions. *Int. J. Biol. Macromol.* **154**, 683–697 (2020). 32194112 10.1016/j.ijbiomac.2020.03.128

[34] Gholizadeh, F. et al. Enhancing wheat resilience to salt stress through an integrative nanotechnology approach with chitosan proline and chitosan glycine. *Sci. Rep.* **15** (1), 11126 (2025). 40169625 10.1038/s41598-025-91496-w PMC11961683

[35] Sheikhalipour, M. et al. Chitosan–selenium nanoparticle (Cs–Se NP) foliar spray alleviates salt stress in bitter melon. *Nanomaterials* **11** (3), 684 (2021). 33803416 10.3390/nano11030684 PMC7999252

[36] Habibi, G. & Aleyasin, Y. Green synthesis of Se nanoparticles and its effect on salt tolerance of barley plants. *Int. J. Nano Dimens*. **11** (2), 145–157 (2020).

[37] Jafari, H. et al. Characterization of pH-sensitive chitosan/hydroxypropyl methylcellulose composite nanoparticles for delivery of melatonin in cancer therapy. *Mater. Lett.* **282**, 128818 (2021).

[38] Bates, L. S., Waldren, R. P. & Teare, I. D. Rapid determination of free proline for water-stress studies. *Plant. Soil.* **39** (1), 205–207 (1973).

[39] Lichtenthaler, H. K. Chlorophylls and carotenoids: Pigments of photosynthetic biomembranes. In *Methods in enzymology*. Vol. 148. Elsevier; pp. 350–382. (1987).

[40] Pál, M., Horváth, E., Janda, T., Páldi, E. & Szalai, G. Cadmium stimulates the accumulation of salicylic acid and its putative precursors in maize (*Zea mays*) plants. *Physiol. Plantarum*. **125**(3), 356–364 (2005).

[41] Livak, K. J. & Schmittgen, T. D. Analysis of relative gene expression data using real-time quantitative PCR and the 2^–∆∆CT^ method. *Methods*. **25**(4), 402–408 (2001). 10.1006/meth.2001.1262 11846609

[42] SAS Institute. *SAS Institute 9.4 language reference: Concepts* (SAS Institute, 2018).

[43] Tang, D. et al. SRplot: A free online platform for data visualization and graphing. *PLoS One*. **18**(11): e0294236 (2023). 10.1371/journal.pone.0294236 PMC10635526 37943830

[44] Iqbal, M. et al. Exogenously applied selenium reduces oxidative stress and induces heat tolerance in spring wheat. *Plant Physiol Biochem*. **94**, 95–103 (2015). 10.1016/j.plaphy.2015.05.012 26057700

[45] Zahra, N. et al. Regulation of photosynthesis under salt stress and associated tolerance mechanisms. *Plant Physiol Biochem*. **178**:55–69 (2022). 10.1016/j.plaphy.2022.03.003 35276596

[46] Yousefi, B. & Karamian, R. Nano-selenium reduced the adverse effects of salinity stress on *Satureja spicigera* (C. Koch) boiss. *J. Med. Plants By-Prod*. **13** (4), 971–981 (2024).

[47] Haghmadad Milani, M. et al. Cerium oxide nanoparticles (CeO_2_ NPs) enhance salt tolerance in spearmint (*Mentha spicata* L.) by boosting the antioxidant system and increasing essential oil composition. *Plants*. **13**(20), 2934 (2024). 10.3390/plants13202934 PMC11510870 39458881

[48] Samynathan, R. et al. A recent update on the impact of nano-selenium on plant growth, metabolism, and stress tolerance. *Plants*. **12**(4):853 (2023). 10.3390/plants12040853 PMC9964709 36840201

[49] Diao, M. et al. Selenium promotes the growth and photosynthesis of tomato seedlings under salt stress by enhancing chloroplast antioxidant defense system. *J. Plant Growth Regul*. **33**, 671–682 (2014).

[50] Hussein, H. A. A., Darwesh, O. M. & Mekki, B. Environmentally friendly nano-selenium to improve antioxidant system and growth of groundnut cultivars under sandy soil conditions. *Biocatal. Agric. Biotechnol*. **18**, 101080 (2019).

[51] Alvarez, M. E., Savouré, A. & Szabados, L. Proline metabolism as regulatory hub. *Trends Plant Sci*. **27**(1), 39–55 (2022). 10.1016/j.tplants.2021.07.009 34366236

[52] Tabassum, M. et al. Chitosan modulated antioxidant activity, inorganic ions homeostasis and endogenous melatonin to improve yield of *Pisum sativum* L. accessions under salt stress. *Sci. Hortic*. **323**:112509 (2024).

[53] Khattabi, D., Sakar, E. & Louahlia, S. Flag leaf tolerance study in Moroccan barley (*Hordeum vulgare* L.) varieties submitted to a severe salt stress. *Biointerface Res. Appl. Chem*. **12**(3), 2787–2799 (2022).

[54] Gharaghanipor, N., Arzani, A., Rahimmalek, M. & Ravash, R. Physiological and transcriptome indicators of salt tolerance in wild and cultivated barley. *Front Plant Sci*. **13**, 819282 (2022). 10.3389/fpls.2022.819282 PMC9047362 35498693

[55] Alkahtani, J. & Dwiningsih, Y. Analysis of morphological, physiological, and biochemical traits of salt stress tolerance in Asian Rice cultivars at seedling and early vegetative stages. *Stresses*. **3**(4), 717–735 (2023).

[56] Nefissi Ouertani, R. et al. Transcriptomic analysis of salt-stress-responsive genes in barley roots and leaves. *Int. J. Mol. Sci*. **22**(15), 8155 (2021). 10.3390/ijms22158155 PMC8348758 34360920

[57] Jadidi, O., Etminan, A., Azizi-Nezhad, R., Ebrahimi, A. & Pour-Aboughadareh, A. Physiological and molecular responses of barley genotypes to salinity stress. *Genes*. **13**(11), 2040 (2022). 10.3390/genes13112040 PMC9690512 36360277

[58] Domokos-Szabolcsy, E. et al. Accumulation of red elemental selenium nanoparticles and their biological effects in *Nicotinia tabacum*. *Plant Growth Regul*. **68**, 525–531 (2012).

[59] Mahboob, W. et al. Salinity tolerance in wheat: responses, mechanisms and adaptation approaches. *Appl. Ecol. Environ. Res*. **21**(6) (2023).

[60] Jabeen, Z., Irshad, F., Hussain, N., Han, Y. & Zhang, G. NHX-type Na+/H+ antiporter gene expression under different salt levels and allelic diversity of HvNHX in wild and cultivated barleys. *Front Genet*. **12**, 809988 (2022). 10.3389/fgene.2021.809988 PMC8902669 35273633

[61] Gholizadeh, F., Mirmazloum, I. & Janda, T. Genome-wide identification of HKT gene family in wheat (*Triticum aestivum* L.): Insights from the expression of multiple genes (*HKT*, *SOS*, *TVP* and *NHX*) under salt stress. *Plant Stress*. **13**, 100539 (2024).

[62] Hazzouri, K. M. et al. Mapping of *HKT1;5* gene in barley using GWAS approach and its implication in salt tolerance mechanism. *Front Plant Sci*. **9**, 156 (2018). 10.3389/fpls.2018.00156 PMC5826053 29515598

[63] Gürel, F., Öztürk, Z. N., Uçarlı, C. & Rosellini, D. Barley genes as tools to confer abiotic stress tolerance in crops. *Front Plant Sci*. **7**, 1137 (2016). 10.3389/fpls.2016.01137 PMC4971604 27536305

[64] Chaudhary, S. et al. Identification and characterization of contrasting genotypes/cultivars for developing heat tolerance in agricultural crops: Current status and prospects. *Front Plant Sci*. **11**, 587264 (2020). 10.3389/fpls.2020.587264 PMC7642017 33193540

[65] Mahalingam, R. et al. Heat and drought induced transcriptomic changes in barley varieties with contrasting stress response phenotypes. *Front. Plant. Sci.* **13**, 1066421 (2022). 36570886 10.3389/fpls.2022.1066421 PMC9772561

